# Fracture behavior of endodontically treated premolar teeth restored with different lithium silicate-based endocrown restorations

**DOI:** 10.1186/s12903-025-07149-x

**Published:** 2025-11-20

**Authors:** Ahmed Shams, Mostafa A. Abdelshafi, Mohammed Talaat Salem

**Affiliations:** 1 Fixed Prosthodontics Department, Faculty of Dentistry, Mansoura University, Mansoura, Egypt; 2 Dental Biomaterials Department, Faculty of Dentistry, Mansoura University, Mansoura, Egypt

**Keywords:** Fracture behavior, Premolar teeth, Endocrown, IPS e.max CAD, Celtra duo, Cerec tessera

## Abstract

**Background:**

This study aimed to assess the fracture behavior of endodontically treated maxillary first premolars restored with different lithium silicate-based endocrown restorations.

**Methods:**

Thirty intact human maxillary first premolars were root canal treated. They were divided into three groups (*n* = 10) based on the CAD/CAM lithium silicate-based material used for endocrown fabrication: Group LD: Lithium disilicate-based ceramic (IPS e.max CAD), Group ZLS: Zirconia-reinforced lithium silicate-based ceramic (Celtra Duo), and Group ALD: Advanced lithium disilicate-based ceramic (Cerec Tessera). After adhesive cementation, all specimens underwent a thermomechanical aging process. Surviving specimens underwent fracture resistance testing, followed by analysis with stereomicroscopy and Scanning Electron Microscopy (SEM). Data were statistically analyzed at P-value ≤ 0.05.

**Results:**

The mean failure load value was significantly higher for Group ALD (1853.89 ± 178.40 N) compared to Group LD (1425.57 ± 156.31 N) and Group ZLS (1364.12 ± 196.34 N). A statistically significant difference in failure mode was detected among tested groups, with more favorable fracture patterns observed within Groups ZLS and ALD.

**Conclusions:**

All studied lithium silicate-based endocrown restorations expressed fracture load values that significantly surpassed the maximum human masticatory force reported for maxillary premolar region supporting their clinical use. Cerec Tessera endocrowns exhibited the best biomechanical behavior among tested restorative materials.

**Clinical trial number:**

Not applicable.

## Background

Restoring endodontically treated teeth (ETT) has been a subject of ongoing debate in dentistry for many years. Following root canal treatment, it is crucial to maintain the residual tooth structure as well as choosing the appropriate restorative materials that not only restore function but also strengthen the tooth [1]. Conventionally, the standard approach for restoring severely damaged teeth has been a crown supported by a post and core system. However, this method can compromise the tooth’s mechanical strength, increasing the risk of root fractures and the possibility of perforation during post space preparation [2]. With advancements in adhesive dentistry along with the innovation of durable high-strength all-ceramic materials, the need for the traditional post and core methodology has become increasingly unnecessary. As a result, endocrown restorations have emerged as an alternate solution for restoring severely damaged teeth [3].

The growing focus on minimally invasive dentistry has introduced new treatment options, such as the monolithic endocrown restoration, which is secured to both the pulp chamber and the cavity margins. This approach achieves macro-mechanical retention through pulpal walls besides micro-mechanical retention via adhesive cementation [4]. Endocrowns have been shown to offer better stability and enhanced fracture strength in comparison with traditional restorations [5]. A systematic review revealed that endocrowns may perform equally well or even outperform conventional treatments, such as those involving intra-radicular posts, direct resin composite, inlay, and onlay restorations. The study reported impressive clinical survival rates above 94% with follow-ups of up to 36 months [6].

A key area of focus is selecting the appropriate restorative material to optimize the behavior of such endocrown restorations [7]. CAD/CAM materials have been developed with enhanced mechanical strength and outstanding optical qualities [8]. The most suitable materials for endocrown manufacturing are reinforced acid-etchable ceramics, as they offer adequate mechanical strength to withstand occlusal forces while providing strong adhesion to the tooth structure [9]. Lithium disilicate-reinforced ceramics, whether pressable or machinable (CAD/CAM), are regarded as the ideal material choice, offering superior mechanical strength, outstanding adhesion to tooth structure, and exceptional esthetic qualities [7].

Among the diverse materials developed for CAD/CAM dental prostheses, lithium disilicate-based glass-ceramics and t derivatives stand out as noteworthy options [10, 11]. Lithium disilicate-based ceramic, branded as IPS Empress 2, made its debut in dentistry in 1998 as an indirect restorative material. It was initially designed for being used with press technology and was succeeded by enhanced versions, comprising IPS e.max Press besides IPS e.max CAD, later on. The CAD version is supplied in a meta-silicate state, consisting of 40% platelet-shaped lithium meta-silicate crystals within a bluish glassy matrix. To achieve the anticipated lithium disilicate structure and shade, a crystallization process is needed, which includes a firing cycle at 840 °C for 25 min [12]. Previous studies have reported the flexural strength of lithium disilicate-based glass-ceramics to range from 300 to 520 MPa, with prosthesis survival rates varying between 96% and 100% over a 3-year period [10].

New restorative materials development focused on combining superior mechanical strength with excellent aesthetic performance, has resulted in the introduction of zirconia-reinforced lithium silicate ceramics (ZLS), which can be utilized with CAD/CAM technologies [13]. A novel dental material with a distinctive chemical composition, which merges the aesthetic advantages of lithium disilicate with the superior mechanical durability of zirconia, owing to the incorporation of 10 wt% zirconium into its glassy structure [14, 15]. In 2013, ZLS was industrialized through a collaboration between Vita Zahnfabrik, Dentsply Sirona, and the Fraunhofer Institute for Silicate Research (Würzburg, Germany). The material was marketed separately under different trademark names: Vita Suprinity and Celtra Duo [13, 16].

An innovative advanced lithium disilicate (ALD) ceramic was developed in 2021 by Dentsply Sirona called Cerec Tessera. It is a high-strength, tooth-colored glass-ceramic block that requires obligatory firing to achieve its maximum strength. ALD ceramic is characterized by a unique microstructure [17]. The manufacturer states that the material is composed of lithium disilicate and virgilite (lithium aluminum silicate) crystals embedded within a zirconia-enriched glass matrix. Additional virgilite crystals grow during the firing process. On one hand, the rod-shaped lithium disilicate crystals enhance tensile strength, helping to prevent crack propagation. On the other hand, the tiny virgilite crystals that form during firing play a significant role in achieving a biaxial flexural strength greater than 700 MPa through enhancing pre-compression stress [17, 18].

There is ongoing debate regarding the appropriateness of endocrowns for restoring severely damaged endodontically treated premolar teeth (ETPT). Lithium disilicate ceramics have been extensively investigated and shown to yield favorable outcomes in endocrown restorations. However, comparative data on the mechanical performance of various lithium silicate-based ceramics in the restoration of severely compromised ETPT remain limited. In this context, this preclinical study was performed to evaluate the fracture behavior of such endodontically treated maxillary first premolar teeth restored with different lithium silicate-based endocrown restorations. The main tested null hypothesis was that there would be no difference in the fracture behavior of the ETPT restored with endocrowns fabricated using different lithium silicate-based ceramic materials.

## Methods

This in-vitro study followed all guidelines set by the Local Research Ethics Committee of the Faculty of Dentistry, Mansoura University, and received approval no. A0101025FP. A power analysis was executed to detect the sample size utilizing the G*Power version 3.0.10, with a sample size of 10 per group, an α error of 0.05, 80% power, and an effect size of 0.25 [19].

### Selection, disinfection, and storage of teeth

Thirty intact, bifurcated maxillary first premolar human teeth with fully developed roots, recently extracted for orthodontic reasons, and with homogenous dimensions and morphology were selected [4, 20, 21]. All teeth were collected after obtaining patient consent from the Oral and Maxillofacial Surgery Department, Faculty of Dentistry, Mansoura University. The initial preparation of the chosen teeth included debridement and cleaning to remove any superficial staining, calculus, and attached soft tissue using an ultrasonic scaler, followed by a polishing step using a prophylactic paste. Teeth with cracks, decay, or existing restorations were excluded [20–22]. Teeth with severely curved or abnormally shaped roots were also excluded [9]. In accordance with the 1993 CDC recommendations (Centers for Disease Control and Prevention, Georgia, USA), the chosen teeth were disinfected for seven days using a 1:10 dilution of 5.25% NaOCl household bleach (Clorox Bleach, Clorox, Cairo, Egypt) [23]. Teeth were kept in distilled water at room temperature for the entire duration of the testing period, with water being replaced on a weekly basis to prevent bacterial growth, serving as a safeguard against dehydration [3, 24].

### Endodontic treatment of teeth

Selected teeth were sectioned and decapitated using a diamond wheel bur (MANI, Tochigi, Japan), parallel to the occlusal surface, 2 mm above the uppermost point of the proximal cementoenamel junction (CEJ) [21, 25, 26]. Teeth underwent endodontic treatment performed by a single operator following a standardized crown-down technique [4, 9]. Canals, 0.5 mm from the apical foramina, were prepared using a Nickel Titanium (NiTi) rotary file system (Dota Fury L60 assorted kit, Dota Medical Instrument, Guangxi, China), according to the manufacturer’s instructions. Root canals were obturated to full working length using the corresponding matched master gutta-percha points and sealed using an epoxy resin-based sealer (Dia-ProSeal, DiaDent, Chungcheongbuk-do, Korea) [21, 27]. Following the obturation procedure and the removal of excess gutta-percha, the access cavity for each tooth was partially filled using a layer of flowable resin composite material (Filtek Z350 XT A2, 3 M ESPE, Seefeld, Germany), according to the manufacturer’s guidelines [28–30].

### Teeth mounting

To facilitate handling of the studied teeth used for fracture resistance testing, each tooth’s roots were vertically embedded along their long axes in pink acrylic resin blocks. Each selected tooth was individually mounted in a cylindrical plastic ring (25 mm depth, 15 mm internal diameter) filled with self-curing acrylic resin material (Acrostone cold cure denture base material, Acrostone, Cairo, Egypt) utilizing a dental laboratory surveyor device (Milling unit BF 2, Bredent, Senden, Germany) [26]. For all selected teeth, the simulated alveolar bone level was marked 1 mm below the CEJ level [20]. An approximately 0.3 mm uniform layer of simulated periodontal ligament (PDL) was created around the roots of all studied teeth following the “Transitional Wax Technique” using a light-body polyvinyl siloxane (PVS) impression material (Perfit Light Body, Huge Dent, China) [26, 31]. All the steps were performed by a single operator.

### Teeth grouping

The selected teeth were assigned into 3 groups (*n* = 10) at random based on the CAD/CAM lithium silicate-based ceramic materials utilized in the fabrication of endocrowns: Group LD: 10 teeth restored with lithium disilicate-based ceramic endocrowns (IPS e.max CAD, Ivoclar Vivadent, Schaan, Liechtenstein), Group ZLS: 10 teeth restored with zirconia-reinforced lithium silicate-based ceramic endocrowns (Celtra Duo, Dentsply Sirona, NC, USA), and Group ALD: 10 teeth restored with advanced lithium disilicate-based ceramic endocrowns (Cerec Tessera, Dentsply Sirona, NC, USA). The CAD/CAM lithium silicate-based ceramic materials utilized in this study are shown in (Table 1).

**Table 1:** The CAD/CAM lithium silicate-based ceramic materials used in this study

Group	Material	Product name	Lot number	Composition	Manufacturer
(LD)	Lithium disilicate glass ceramic	IPS e.max CAD	Y15163	57–80% SiO_2_, 11–19% Li_2_O, < 13% K_2_O, < 5% MgO, < 5% Al_2_O_3_, < 11% P_2_O_5_, < 8% ZrO_2_, < 8% ZnO, < 0.8% coloring oxides	Ivoclar Vivadent, Schaan, Liechtenstein
(ZLS)	Zirconia-reinforced lithium silicate ceramic	Celtra Duo	16,012,706	58% SiO_2_, 18.5% Li_2_O, 10.1% ZrO_2_, 5% P_2_O_5_, 1.9% Al_2_O_3_, 2%CeO_2_, 1% Tb_4_O_7_	Dentsply Sirona, NC, USA
(ALD)	Advanced lithium disilicate ceramic	Cerec Tessera	16,018,858	57–80% SiO_2_, 11–19% Li_2_O, 0–13% K_2_O, 0–11% P_2_O_5_, 0–8% ZrO_2_, 0–8% ZnO, 0–12% other oxides	Dentsply Sirona, NC, USA

### Endocrown tooth preparation

In this study, a standardized endocrown preparation was conducted on all the natural teeth using the dental laboratory surveyor device (Milling unit BF 2). All selected teeth received butt-margin endocrown preparations by the same operator with the help of 5x-magnification dental loupe [32]. At this stage, the occlusal reduction had already been performed 2 mm occlusal to the proximal CEJ using a diamond wheel bur aligned with the tooth’s long axis and kept parallel to the occlusal plane. Internally, preparation was limited to removing undercut areas while preserving the pulp chamber’s anatomical structure, ensuring smooth and rounded interior line angles. An 80 μm-grain size cylindrical-conical diamond bur (8113R, Intensiv Inlay Set, Intensiv SA, Montagnola, Switzerland) with rounded corners and a taper angle of 6° was used for axial preparation along the tooth’s long axis, without undue pressure, leaving only about 1 mm of the flowable composite seal [33, 34].

A 25 μm-grain size cylindrical-conical diamond bur (3113R, Intensiv Inlay Set, Intensiv SA, Montagnola, Switzerland) with the same taper and rounded corners was used for internal cavity finishing. For butt-margin finishing, a diamond bur (3113NR, Intensiv Inlay Set, Intensiv SA, Montagnola, Switzerland) of the same taper and particle size as the one used for internal cavity finishing, but with a larger diameter was used. It was carefully guided around the entire circumference of the cervical band (butt-margin) using the same diamond wheel orientation to eliminate irregularities and create a smooth, polished surface [33]. Finally, the features of the endocrown preparation were re-evaluated and visually examined to confirm that the original pulp chamber structure was preserved. This was done through probe palpation to confirm a smooth finish without undercuts, followed by measurement with a digital caliper to verify the preparation measurements: 4 mm depth, 5 ± 0.2 mm buccopalatal width, 3 ± 0.2 mm mesiodistal width, and 2 ± 0.2 mm thickness of the circular axial wall [8].

### Endocrowns fabrication

According to teeth grouping, a total of thirty endocrowns were designed and milled utilizing a CAD/CAM system: 10 IPS e.max CAD (LT A2/C14) endocrowns for Group LD, 10 Celtra Duo (LT A2/C14) endocrowns for Group ZLS, and 10 Cerec Tessera (MT A2/C14) endocrowns for Group ALD. The CAD/CAM process chain, which consisted of scanning, designing and milling phases, was followed. Each prepared tooth was scanned with a high-precision 3D optical scanner (Identica Hybrid, Medit Dental, Seoul, Korea) with scan accuracy of ± 7 μm. A specialized software (colLab Scan, v2.0.0.4, Medit Dental, Seoul, Korea) was employed to finalize the scanning procedure, ensuring the generation of high-resolution scan data.

**Fig. 1:**
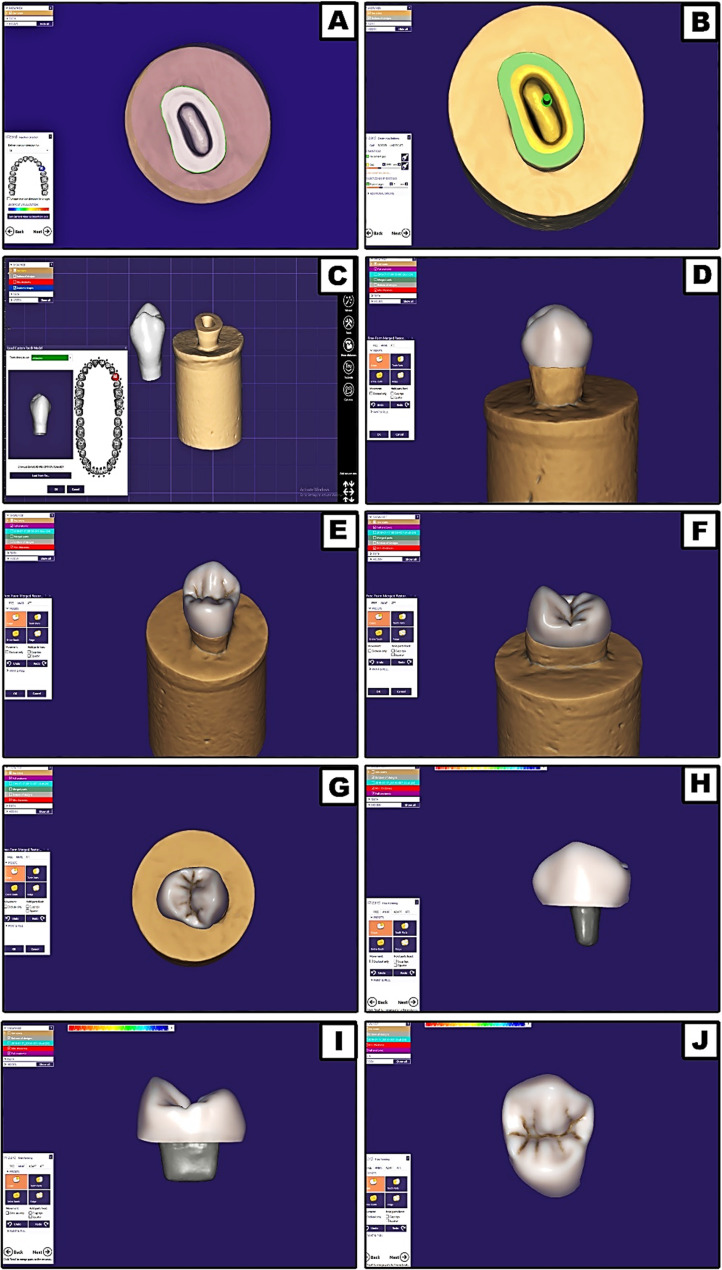
Designing phase for endocrowns fabrication; (A) Margin line defining, (B) Determination of cement gap thickness (50 μm) and its distance from margin (1 mm) with endocrown path of insertion, (C) Determination of a standardized endocrown morphology (alternative reference model was selected), (D-G) The selected model tooth auto-adaptation, and (H-J) The finished full anatomical master endocrown design

Each scanned tooth’s endocrown restoration was designed with the aid of dental CAD software (DentalDB 2.2 Valletta, exocad, Darmstadt, Germany). It included margin line defining, determination of cement gap thickness (50 μm) [7, 9] and its distance from margin (1 mm) and the endocrown path of insertion. To standardize the morphology of the endocrowns across all study teeth, a model of maxillary first premolar was selected from the software database library as the master reference model and applied to all teeth. The model was then automatically adapted to the specific characteristics of each tooth (Fig. 1). Based on the applied master reference model of this maxillary first premolar (Alternative model), the endocrown occlusal thickness for all tested groups was designed standardized at 6.0 mm buccally (measured from buccal cusp tip to butt-margin level), 5.0 mm palatally (measured from palatal cusp tip to butt-margin level), and 3.5 mm proximally (measured from proximal marginal ridge to butt-margin level).

The finalized endocrown design for each scanned tooth was milled using a 5-axis wet milling machine (CORiTEC 250i touch, imes-icore GmbH, Eiterfeld, Germany) from the corresponding block material. The post-milling crystallization and glazing procedures were performed for LD and ALD groups in accordance with the manufacturer’s instructions using a compatible ceramic furnace with controlled long-term cooling and vacuum functions (Programat P500, Ivoclar Vivadent, Schaan, Liechtenstein). Regarding ZLS endocrowns, the “mill, polish, fire” processing protocol was used. After milling, the endocrowns were tested to ensure that they fit properly on each corresponding tooth. They were then polished using diamond stones and rubber polishers (Celtra Polishing kit, Dentsply, Germany) at low speed and with minimal pressure and finally firing according to manufacturer’s instructions.

### Endocrowns cementation

#### Surface treatment of tooth preparation

For the cementation of the endocrown restorations, the cleaned prepared teeth were etched for 15 s using a 37% phosphoric acid etchant (Meta Etchant, Meta Biomed Co., Ltd., Cheongju, South Korea), followed by thorough water rinsing and gentle air drying. A thin layer of a universal dental adhesive (G-Premio BOND, GC Corporation, Tokyo, Japan) was then applied, air-thinned, and light-polymerized for 10 s using a type of LED light-curing unit (Elipar DeepCure-S, 3 M ESPE Dental, MN, USA) with a power intensity of approximately 1,470 mW/cm² and a wavelength range of 430–480 nm. The light output was checked with a radiometer before each bonding procedure.

#### Surface treatment of endocrown restoration

The clean, dry fitting surfaces of all endocrown restoration`ns were first treated through application of 9.5% hydrofluoric acid etching gel (Porcelain Etchant, Bisco, IL, USA) following the manufacturers’ recommendations. Then, these surfaces were silanized using a single-component silane coupling agent (Porcelain Primer, Bisco, IL, USA), which was left in place for 30 s and carefully blown for 5 s with a light stream of air, as recommended by the manufacturer.

#### Application of resin luting cement

**Fig. 2:**
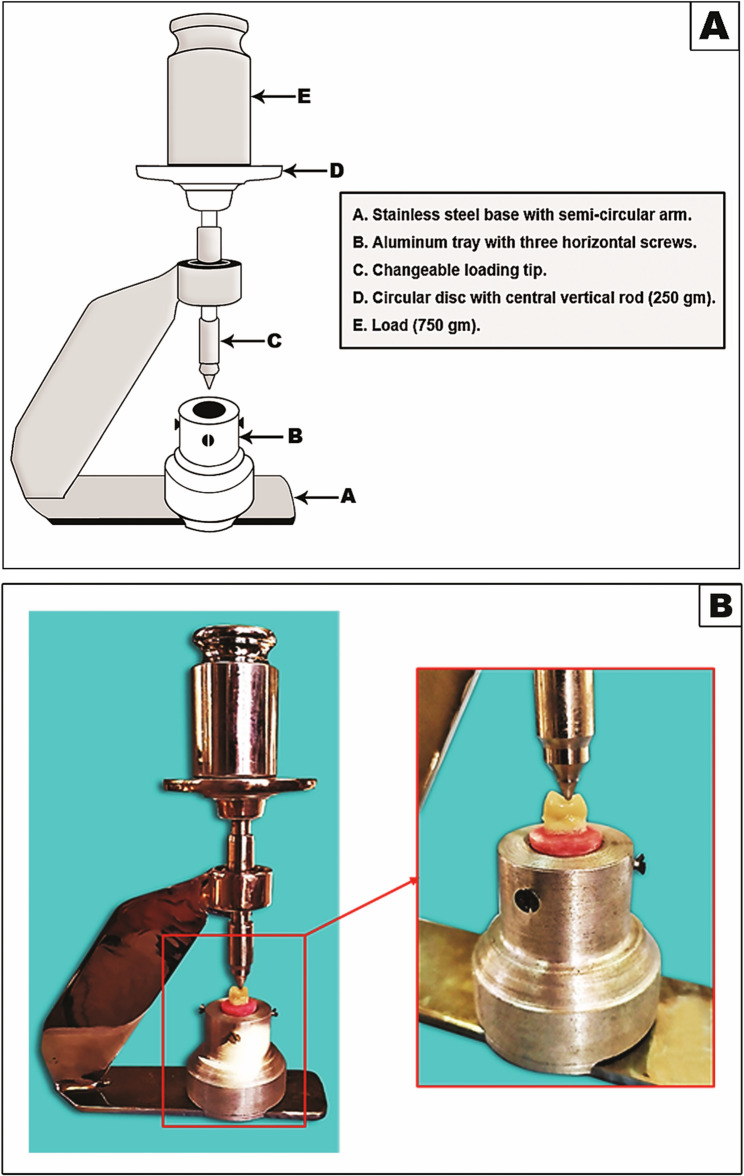
Using of a specially-designed custom-made loading device to standardize the load applied during the endocrowns cementation procedure; (A) Illustrated schematic diagram of the used device and (B) The specimen in its secured position while the load is applied

A dual-cured, radiopaque, syringeable adhesive resin cement (G-CEM LinkForce; A2 shade, GC Corporation, Tokyo, Japan) was utilized to cement each endocrown onto its corresponding tooth. Each endocrown, held on an adhesive plastic pick-up stick (OptraStick, Ivoclar Vivadent, Schaan, Liechtenstein), was carefully positioned on its respective tooth with light finger pressure to ensure initial proper seating. The specimens were subsequently placed into a specially-designed custom-made loading device in order to standardize the force applied during the cementation procedure [9, 22, 35] (Fig. 2). A static axial load of 1 kg was exerted vertically at a right angle to the occlusal surface of the endocrown and sustained for 30 s prior to light curing [4, 36].

In line with the manufacturer’s guidelines, initial spot curing of the excess cement was carried out for 2–3 s at each margin to allow removal of this excess cement using a scaler [22, 35]. After cleaning of the trace cement remnants, light-curing step was completed for up to 40 s per margin. The light output was verified using a radiometer prior to each use. All endocrown restorations in each group were finished along the marginal cementation lines using fine-grit diamond burs (30 μm; Mani Inc., Tochigi, Japan) under water cooling to remove surface irregularities and ensure smooth margins. Polishing was subsequently performed with the Shofu Ceramisté polishing kit (Shofu Dental, Kyoto, Japan) in a multi-step sequential manner according to the manufacturer’s instructions [4, 20, 24]. Finally, all cemented specimens were stored and preserved in distilled water at 37 °C in an incubator for 24 h prior to testing [22, 37].

### Fatigue testing

#### Thermal cycling testing (Thermal aging)

All cemented specimens underwent an"artificial thermomechanical aging" protocol. Utilizing a thermocycler device (Thermo Scientific, Thermo Fisher Scientific, MA, USA), the specimens underwent 10,000 cycles alternating between 5 ± 1°C and 55 ± 1°C, with a 30 seconds dwell time within each water bath along with a transfer time of 5 seconds, simulating roughly 1 year of clinical function [7, 38–40].

#### Dynamic loading testing (Mechanical aging)

All cemented specimens underwent 240,000 loading cycles utilizing a chewing simulator device (CS-4.4, SD Mechatronik, Feldkirchen-Westerham, Germany), designed to simulate 1 year of oral function. This cyclic loading was applied at a frequency of 1.6 Hz with a load magnitude of 50 N. The specimens were loaded in distilled water at 37 °C using a steatite-ceramic, ball-shaped antagonist, 6 mm in diameter. The ball was positioned at the central point of the occlusal surface, engaging both the buccal and palatal cusps, while being aligned parallel to the tooth’s long axis [19, 29, 30].

### Fracture testing

#### Fracture resistance evaluation (Failure load)

Following fatigue testing, the surviving specimens underwent fracture resistance evaluation utilizing a universal testing machine (model 3365, Instron Industrial Products, MA, USA). The compressive load was applied axially and centrally with the use of a 5 kN load cell, equipped with a 6 mm-diameter stainless steel (SS) ball-shaped loading piston, operating at a crosshead speed of 0.5 mm/min, till the specimen underwent plastic deformation or fracture. The maximum load required to induce a fracture was measured in newton (N) [7, 19, 25, 35].

#### Fractography (Failure mode)

A pre-calibrated stereomicroscope (Olympus SZ 60, Tokyo, Japan) at a magnification of 40x was used to qualitatively examine the fractured specimens. Following the evaluation of all specimens by a consensus of three examiners, the failure modes were identified and categorized based on the established criteria in Table 2 [7, 19, 30]. Scanning electron microscopy (SEM) (JEOL JSM-5500LV SEM, JEOL Ltd, Tokyo, Japan) was used to evaluate fractured specimens, representative of each failure mode across all groups. Micrographs of the fracture surfaces were captured and recorded at magnifications of 20x and 40x with a working distance of 10 mm, depending on the target region. The scanning micrographs were evaluated to determine the failure mode by systematically mapping the fracture origin(s) and the crack propagation direction within all studied groups [3, 30, 37].

**Table 2:** Classification of the failure modes

Type	Failure mode	Description	Prognosis
I	Adhesive failure	Debonding of the endocrown restoration without fracture.	Non-catastrophic/repairable/favorable
II	Cohesive failure	Fracture of the endocrown restoration without displacement (no loss of adhesion).	
III	Cohesive-Adhesive failure	Fracture of the endocrown restoration with displacement (loss of adhesion).	
IV	Complex fracture above the CEJ	Fracture of the endocrown restoration/tooth complex above the CEJ.	
V	Complex fracture below the CEJ	Fracture of the endocrown restoration/tooth complex below the CEJ, which requires tooth extraction.	Catastrophic/non-repairable/unfavorable

### Statistical analysis

The acquired data were tabulated, coded, and analyzed using SPSS statistical software (version 22, IBM, NY, USA). Quantitative data were obtained as mean ± standard deviation following testing for normality with the Shapiro-Wilk test. Qualitative data were designated using numbers and percentages. The significance of difference was evaluated using the one-way ANOVA test, Post hoc Tukey test for pairwise comparison, and Monte Carlo test. The statistical significance of the reported results was set at *P* ≤ 0.05.

## Results

All studied specimens endured the artificial thermo-mechanical aging technique without exhibiting any noticeable signs of premature failure, achieving a 100% survival rate across all groups. The average failure load values for Group LD, Group ZLS and Group ALD were 1425.57 ± 156.31 N, 1364.12 ± 196.34 N, and 1853.89 ± 178.40 N, respectively. The highest mean value of load-to-failure was recorded for premolar teeth restored with advanced lithium disilicate based ceramic endocrowns, while the lowest mean value was measured for the teeth restored with zirconia-reinforced lithium silicate based ceramic endocrowns. However, all values were significantly higher than the average masticatory force measured in the maxillary premolar region (450 N) [7, 19, 38] (Table 3).

One-way ANOVA test demonstrated a statistically significant difference between tested groups in terms of the mean failure load values (*p* < 0.001*). Post hoc Tukey test was conducted for pairwise comparison of fracture load values among tested groups. It demonstrated that there were statistically significant differences among all tested groups except between LD and ZLS groups. In other words, a non-significant statistical difference only resulted between the mean fracture load values when premolar teeth were restored with both IPS e.max CAD and Celtra Duo endocrowns (Table 3).

**Table 3:** Descriptive statistics (Mean ± SD) with One-way ANOVA test (F) for comparison of fracture load values in Newton (N) and Monte Carlo test (MC) for comparison of failure modes among tested groups

Group	n	Failure Load				Failure Mode							
		Mean	SD	*F*	P	**Favorable**					**Unfavorable**		MC
						I	II	III	IV	Total %	V	Total %	p
(LD)	10	1425.57^a^	156.31	8.23	< 0.001*	0	0	0	0	0%	10	100%	0.001*
(ZLS)	10	1364.12^a^	196.34			0	0	2	2	40%	6	60%	
(ALD)	10	1853.89^b^	178.40			0	0	1	2	30%	7	70%	

^abc^ for significance of Post hoc Tukey test: Mean values (± SD) with same superscripted letters represent non-significant difference and different superscripted letters represent significant one

*SD* Standard Deviation. Group *LD* lithium disilicate-based ceramic endocrowns, Group *ZLS* zirconia-reinforced lithium silicate-based ceramic endocrowns, and Group *ALD* advanced lithium disilicate-based ceramic endocrowns

*Significance at p-value ≤ 0.05

**Fig. 3:**
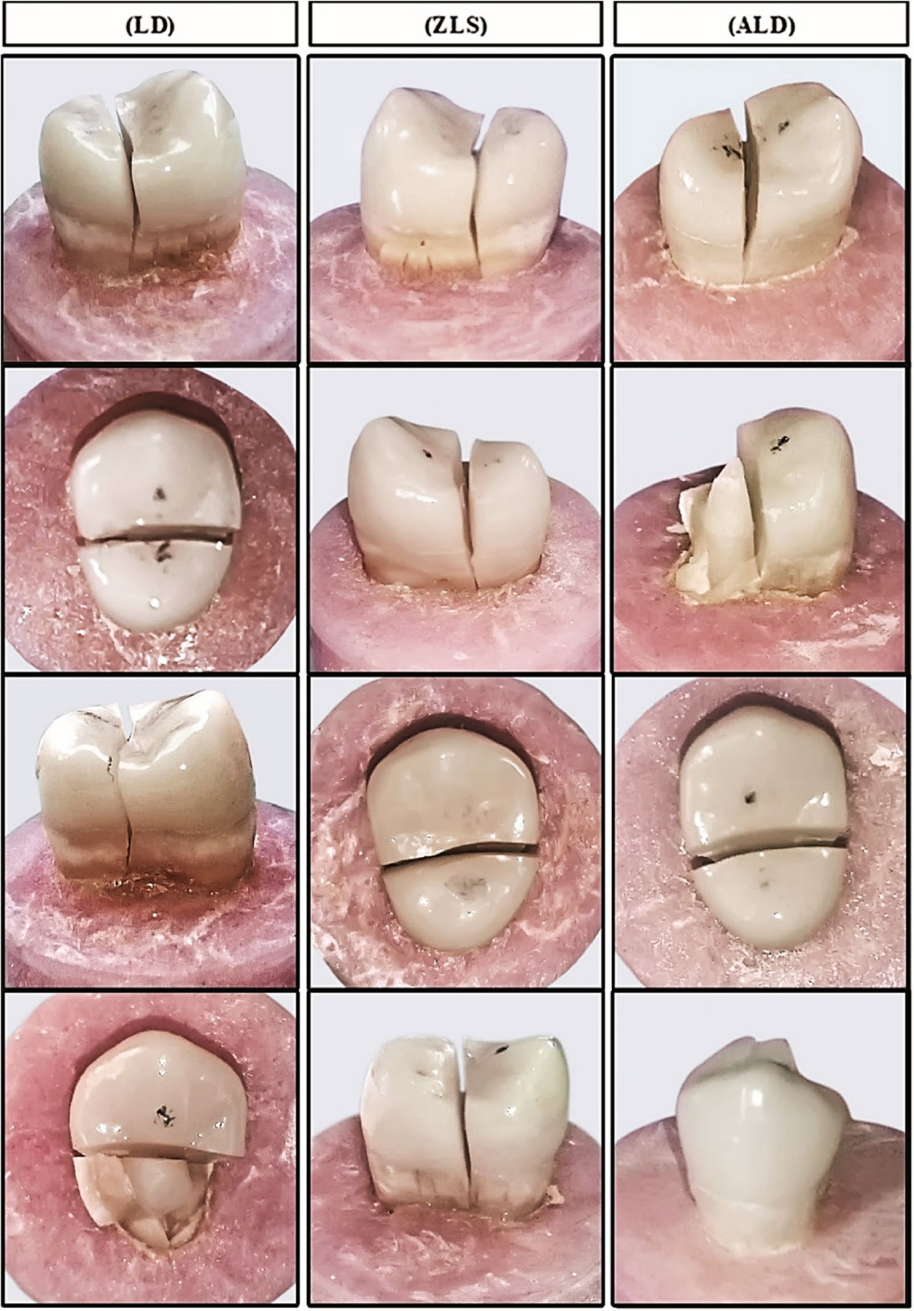
Representative stereomicroscopic images for failure mode assessment of the fractured specimens for all tested groups

Stereomicroscopic analysis (Fig. 3) showed that all studied specimens of LD Group displayed only the undesirable fracture pattern (100% Type V), together with the IPS e.max CAD endocrown/tooth complex fracturing beneath the CEJ. The majority of specimens displayed a catastrophic vertical fracture while a few had fractures in multiple fractured pieces. For ZLS and ALD groups, the tested specimens showed a more favorable pattern of fractures. They expressed 40% and 30% favorable fractures (Type III and Type IV), respectively. Monte Carlo test demonstrated a statistically significant difference among tested groups considering the failure mode (*p* = 0.001) (Table 3).

**Fig. 4:**
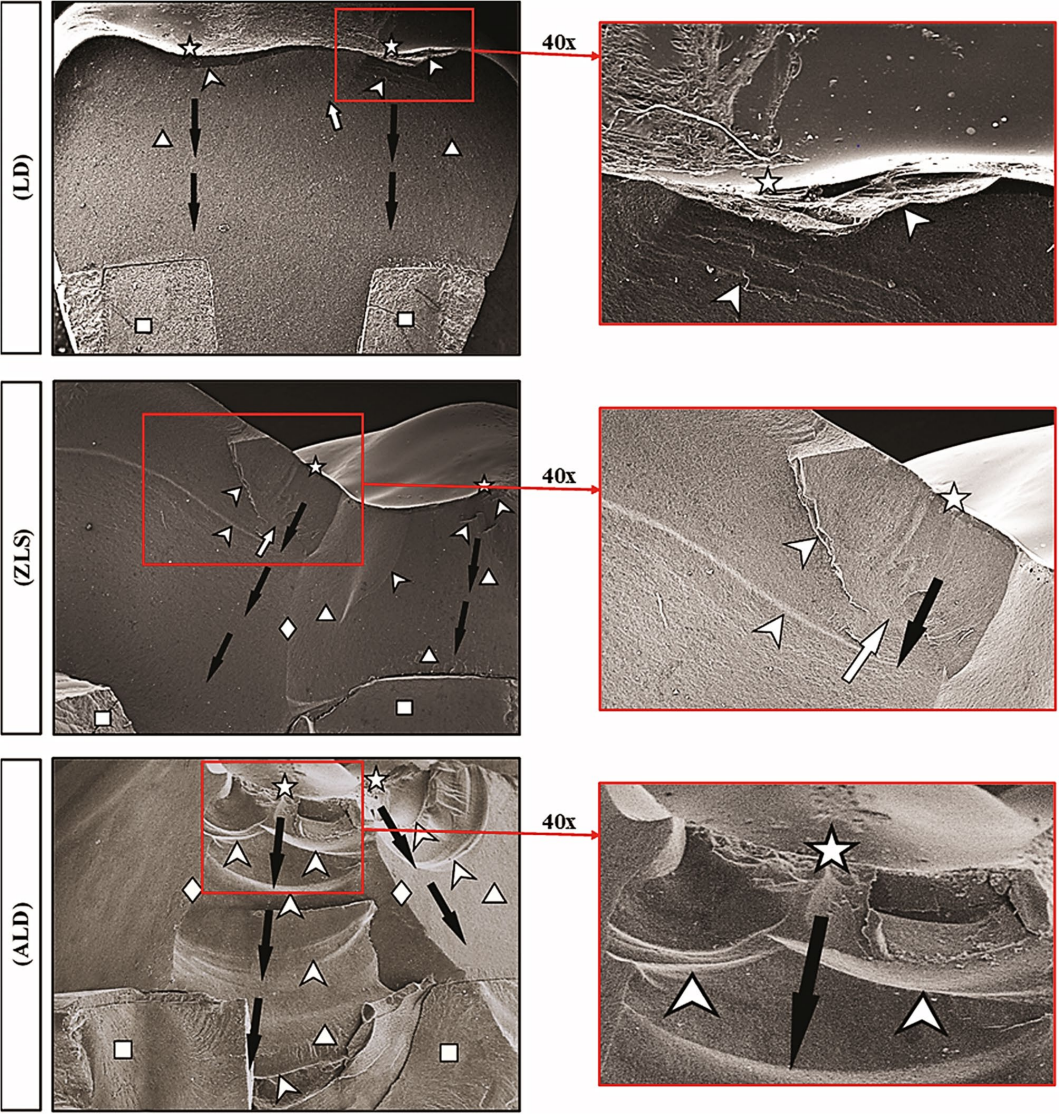
Representative SEM images (20x & 40x) for fractographic analysis of the fractured specimens for all tested groups; Star: Origin of the fracture at the occlusal surface loading area. Triangle: Hackle lines that are clearly visible indicating direction of crack propagation (DCP) represented by “Black arrows”. Arrowhead: Arrest lines representing attempts against crack progress (diffused for ZLS and ALD groups). White arrow: Small internal chip limited with arrest lines. Diamond: Compression curl indicating the CP ends and begins in another direction before total fracture. Square: Fracture of the endocrown/tooth complex

SEM fractographic analysis for all tested groups (Fig. 4) revealed that the primary origin of the fracture in all specimens was located on the occlusal surface, directly beneath the main contact loading area during fracture resistance test. The fracture was then advanced corono-apically with the existence of hackle lines and arrest lines demonstrating the direction and the path of crack propagation. Some specimens exhibited supplementary minor events (secondary fracture origins) on the occlusal surface. Tooth fracture was demonstrated in all examined specimens of LD Group. Diffused, as well as distinct, main arrest lines (MAL) coupled with noticeable irregularities were identified through fractured specimens of Groups ZLS and ALD. Compression curls (CC), indicating that the propagated crack terminates and starts in another direction before overall fracture, were also identified.

## Discussion

The current study was performed to assess the mechanical performance and fracture behavior of endodontically treated maxillary first premolar teeth restored with different lithium silicate-based endocrown restorations. The selected teeth were restored with a lithium disilicate-based ceramic material (IPS e.max CAD), a zirconia-reinforced lithium silicate-based ceramic material (Celtra Duo), and an advanced lithium disilicate-based ceramic material (Cerec Tessera), using the CAD/CAM approach. The fracture resistance testing and fractography results indicated a statistically significant difference between the groups tested considering both the mean failure load value and the failure mode. Accordingly, the main tested null hypothesis that there would be no difference in the fracture behavior of the ETPT restored with endocrowns fabricated using different lithium silicate-based ceramic materials was rejected.

In this study, maxillary first premolars were chosen because the effectiveness of endocrowns in restoring endodontically treated premolars still requires further validation [20]. Previous studies have shown considerable disagreement concerning using endocrowns as a viable restorative option for premolars. A few studies have indicated that endocrowns for maxillary premolars offer higher fracture resistance compared to traditional restorations, making them a conservative, aesthetic, and clinically viable restorative option [22, 39–41]. On the other hand, others have reported that endocrowns seemed inadequate and failed more often when fixed to premolars compared with conventional crowns. This is probably attributed to their smaller adhesive area and increased crown height in comparison to molars. Furthermore, premolars experience more horizontally directed (non-axial) forces than molars, which could influence their fracture resistance as well [21, 39, 42].

In the present study and resembling most documented study designs, a resilient light-body layer of polyvinyl siloxane impression material using the ‘’Transitional Wax Technique’’ was applied around all teeth roots to artificially simulate the periodontal ligament during in-vitro testing. The presence of the periodontal ligament (PDL) layer acts as a shock absorber, enabling for a more accurate emulation of tooth movement with balanced stress distribution throughout the artificial PDL material [26, 31]. Also, following most of the literature, rigid acrylic resin material was utilized to mimic the supporting human bone, as it possesses an elastic modulus comparable to that of bone [7, 29, 35].

Specimens were subjected to artificial thermomechanical aging to mimic the oral environment besides gaining a deeper understanding of how the studied materials perform under intraoral conditions as an initial screening test. In 1994, the International Organization for Standardization (ISO TR 11405) recommended that a thermal cycling protocol between 5 °C and 55 °C serves as an accelerated aging test. This process mimics the physiological temperature fluctuations in the oral cavity caused by hot and cold beverages. It is suggested that thermal cycling be conducted within a range of 3,000 to 100,000 cycles, and it is proposed and accepted that 10,000 cycles mimic one year of oral life based on the hypothesis that such thermal cycles likely take place between 20 and 50 times per day [19, 43, 44]. In addition, it was described that a clinical wearing period of five years can be simulated by 1,200,000 chewing cycles. Accordingly, all specimens underwent 240,000 loading cycles via a chewing simulator machine to simulate a wearing period of one year [7, 19, 29].

All of the evaluated specimens successfully endured the one-year artificial thermomechanical aging protocol with no observable signs of early failure (chipping, crack, debonding, or fracture), with a 100% survival rate for all groups. Group LD endocrowns demonstrated a remarkable survival rate, which is consistent with other studies testing IPS e.max CAD endocrowns for maxillary premolars, where all specimens survived aging without any detectable damage [9, 19, 20].

The highest occlusal forces are concentrated in the molar region (especially the first molar), which can reach as much as 900–1000 N in cases of extreme parafunctional bruxism [7, 19]. This means that, in spite of the statistically significant differences, the detected mean fracture loads for all groups at the moment of fracture under axial loading (1425.57 ± 156.31 N for Group LD, 1364.12 ± 196.34 N for Group ZLS, and 1853.89 ± 178.40 N for Group ALD) not only exceeded the normal masticatory forces in the maxillary premolar region (450 N) [7, 19, 38] and the force during clenching (660 N) [7, 21], but also surpassed the peak masticatory forces referenced in the literature.

The results of the contemporary study demonstrated that Cerec Tessera group of endocrowns had the highest mean fracture load among the tested groups of endocrowns. This finding correlates with the study by Kassem et al., which found that Cerec Tessera endocrowns had the highest mean fracture strength, followed by IPS e.max CAD endocrowns with a statistically significant difference [45]. Moreover, IPS e.max CAD endocrowns exhibited a higher value of fracture load compared to Celtra Duo endocrowns. This aligns with the findings of the study by Atiya and Kadhim, which assessed the fracture strength of endocrown restorations milled from various CAD/CAM materials and bonded to maxillary premolars [46]. In addition, the Celtra Duo endocrowns tested in this study had a mean fracture load value of 1364.12 ± 196.34 N which is nearly similar to the mean value of 1377 ± 307 N that was reported by Heikal et al. [47].

In addition to the analysis of the fracture load, it is also relevant to understand the fracture mode to evaluate the tooth for fractured structure retrievability [36]. This study highlighted a statistically significant difference between the tested groups in terms of failure mode (*p* = 0.001). All tested specimens of Group LD showed only the unfavorable, non-repairable, or catastrophic fracture pattern (100% Type V). In contrast, only 60% of Group ZLS specimens and 70% of Group ALD specimens exhibited the unfavorable fracture pattern, while 40% and 30% of specimens expressed a type of favorable, repairable, or non-catastrophic fracture for both groups, respectively.

A limited number of studies have been conducted to assess the fracture resistance of endodontically treated maxillary premolar teeth restored with endocrown restorations. Group LD specimens showed a 100% catastrophic fracture pattern, which is consistent with other studies testing IPS e.max CAD endocrowns for maxillary premolars, where nearly all specimens showed an unfavorable fracture mode that extended into the root [9, 20]. In particular, our findings may be expressed in compliance with the results of Rocca et al., study, where the majority of IPS e.max CAD endocrowns fractured, exhibiting a mesiodistal vertical split, which caused the restoration to break and the fracture to catastrophically extend into the root [20]. Other maxillary premolar studies that resulted in more favorable fractures; 20% [21] and 60% [48], were proceeded with several differences in the study designs.

The high fracture load values obtained for all groups may be attributable to numerous variables. The endocrown’s ceramic occlusal section thickness may impact the performance of the tooth/endocrown complex, with greater occlusal thickness leading to higher fracture resistance. The fracture load measurements of glass-ceramic endocrowns were dependent on specimen thickness, indicating that their fracture resistance improves with an increase in occlusal thickness [22]. Additionally, it can be expected that enhanced restorative material adhesion results in improved stress distribution throughout the system, which in turn enhances fracture resistance [21].

Differences in composition, manufacturing techniques, crystals content and crystallization parameters between different CAD/CAM lithium silicate-based ceramic materials are reflected not only in the different microstructure of these materials, but in their mechanical and clinical performance as well [49]. The difference between materials in terms of composition and microstructure influences each material’s flexural strength and fracture toughness [50]. In this study, Celtra Duo endocrowns presented the lowest mean fracture load value, that may be related to the decreased values of flexural strength and fracture toughness [46].

To save time for clinicians, new CAD/CAM restorative materials have been developed that do not need heat treatment to reach sufficient strength. One such example is Celtra Duo, which is a zirconia-reinforced lithium silicate CAD/CAM material that can optionally be subjected to heat treatment. Even though heat treatment is not required for crystallizing the material, the flexural strength of fired Celtra Duo has been shown to be significantly higher than that of the unfired, as-milled material [35]. This could explain the higher average fracture resistance observed when employing the “mill, polish, fire” processing strategy to manufacture the Celtra Duo endocrowns, as opposed to other studies that used the unfired version [46].

Despite Celtra Duo being reinforced with zirconia, it contains only 10 wt% dissolved zirconia to strengthen the glass matrix. The manufacturer claims that the zirconia particles help reinforce the ceramic structure by interrupting cracks. However, the amount of crystal filler in the material is another factor that significantly impacts the strength of the ceramic. As stated by the manufacturer, crystallized Celtra Duo has a lower crystal filler volume (36% of lithium disilicate and lithium silicate) in contrast to crystallized IPS e.max CAD, which has 70% lithium disilicate by volume [35]. This may be the reason behind the lower mean value of fracture load measured for Celtra Duo endocrowns when compared with IPS e.max CAD endocrowns.

The significantly higher fracture load value for Cerec Tessera endocrowns could be attributed to the different microstructure of this ceramic material compared to that of the other tested materials. According to the manufacturer, Cerec Tessera has a special microstructure in which 0.5 μm long lithium disilicate crystals are embedded within a glassy matrix, along with 0.2–0.3 μm platelet like lithium aluminum silicate crystals (Li_0.5_Al_0.5_Si_2.5_O_6_) referred to as virgilite. During firing of the crowns, more virgilite crystals are formed. These crystals, together with the lithium disilicate crystals, might generate high tensile strength and stop crack propagation, which could increase the endocrowns’ fracture strength. On the other hand, there was no significant statistical difference in the mean fracture load values between IPS e.max CAD and Celtra Duo groups of endocrowns. These could be rendered to the similar crystal content of both materials [45].

Furthermore, the fitting precision of restorations has been demonstrated to be a key factor in the long-term clinical success of these restorations [51]. The butt-margin design utilized in this investigation offers a configuration that avoids the complexity of a ferrule or thin margins, reducing the limitations of the milling bur in accurately reproducing endocrown’s intaglio surface. This design also allows for the smooth escape of excess resin cement, ensuring appropriate seating besides internal marginal fit of all endocrowns while minimizing marginal gaps [35, 52]. From a biomechanical standpoint, axial compressive stresses that were applied during testing along the long axis of the tooth, are resisted and dispersed over the stable butt-margin, which was prepared perpendicularly to this long axis and parallel to the occlusal plane [22].

Restorative materials with a high modulus of elasticity tend to transfer greater amounts of stress to the deeper tooth structure parts, which can lead to harmful effects [1]. As a result, a stiffer endocrown with reduced elasticity, counteracting tooth natural function, generates zones of shear and tension near the interface between the dentin and the cemented restoration. Debonding or fractures may develop from these stress concentrations, which vary in intensity according to the stiffness discrepancies between the tooth and the cemented endocrown [26]. This fact verifies our findings, since unfavorable catastrophic fractures extending to the root were dominated for the tested rigid glass ceramic materials.

All evaluated specimens of Group LD displayed only the Type V unfavorable fracture pattern, according to the stereomicroscopic analysis, while ZLS and ALD groups expressed only 60% and 70% unfavorable fractures, respectively. Moreover, the SEM fractographic analysis of all tested groups revealed that fractures primarily originated at the loading contact point, with crack propagation in a corono-apical direction. Some specimens exhibited secondary origins on the occlusal surface. Finite Element Analysis (FEA) and Weibull Analysis studies, which assess both the stress distribution patterns and failure probability, can further explain these findings in ETPT restored with various endocrown systems [19, 53].

With all efforts done in this in-vitro study to simulate the clinical situation, it has limitations that should be considered when interpreting the findings. First, the controlled laboratory setting cannot fully replicate the complex and dynamic conditions of the oral environment, such as variations in chewing forces, thermal changes, and moisture. Although thermomechanical aging was applied, it may not entirely simulate the long-term functional stresses that restorations undergo in-vivo. The unidirectional axial load applied does not reflect the multidirectional forces present clinically [34, 50]. Additionally, the use of a standardized endocrown preparation design does not reflect the diversity of clinical situations, where the amount of remaining tooth structure and cavity configuration can vary significantly. The study was also limited to maxillary first premolars, which may affect the generalizability of the results to other posterior teeth. Finally, the relatively small sample size may influence the statistical power of the findings. Therefore, future research should prioritize evaluating the performance of the tested endocrowns with various designs and materials under different loading conditions before initiating long-term clinical studies to either confirm or refute the findings of this study.

## Conclusions

Considering the conditions and outcomes of this in-vitro study, the following conclusions were reached:

1. All studied lithium silicate-based endocrown restorations expressed fracture load values significantly surpassed the maximum human masticatory force reported for maxillary premolar region supporting their clinical use.
2. Among tested restorative materials, lithium disilicate-based ceramic (IPS e.max CAD) endocrowns showed the worst fracture behavior in terms of fracture mode, while zirconia-reinforced lithium silicate-based ceramic (Celtra Duo) endocrowns had the lowest fracture load.
3. Advanced lithium disilicate-based ceramic (Cerec Tessera) endocrowns exhibited the best biomechanical behavior among tested restorative materials.

## Data Availability

The datasets generated and/or analyzed during the current study are available from the first author “Ahmed Shams” upon request.

## Abbreviations

LD: Lithium disilicate-based ceramic
ZLS: Zirconia-reinforced lithium silicate-based ceramic
ALD: Advanced lithium disilicate-based ceramic
SEM: Scanning electron microscope
ETT: Endodontically treated teeth
ETPT: Endodontically treated premolar teeth
CAD/CAM: Computer-aided design/computer-aided manufacturing
CDC: Centers for disease control and prevention
CEJ: Cementoenamel junction
NiTi: Nickle titanium
PDL: Periodontal ligament
PVS: Polyvinyl siloxane
SS: Stainless steel
N: Newton
MAL: Main arrest lines
CC: Compression curls
ISO: International organization for standardization
FEA: Finite element analysis
SD: Standard deviation
